# Intraoperative cystoscopy for urinary tract complications during robotic gynecological hysterectomy surgery

**DOI:** 10.1007/s11701-025-02407-0

**Published:** 2025-05-28

**Authors:** Chikako Nagata, Shintaro Yanazume, Yusuke Kobayashi, Mika Fukuda, Mika Mizuno, Shinichi Togami, Hiroaki Kobayashi

**Affiliations:** Department of Obstetrics and Gynecology, Faculty of Medicine, Kagoshima University, 8-35-1 Sakuragaoka, Kagoshima, 890-8520 Japan

**Keywords:** Gynecology, Robotic surgery, Cystoscopy, Urinary tract, Bladder, Hysterectomy

## Abstract

The effectiveness of cystoscopy in reducing urinary tract complications during robotic gynecologic surgery is poorly documented. Since the introduction of robotic surgery at our institution, cystoscopy has been consistently employed as a standard practice, and its usefulness was investigated. This retrospective study evaluated the utility of routine cystoscopy in patients who underwent robotic surgery between February 2017 and April 2024. The outcome was the detection rate of bladder and ureteral complications. Indigo carmine was injected intravenously while suturing the post-hysterectomy vaginal stump. Light permeation of the bladder wall was visually assessed intra-abdominally. Any leakage of the indigo carmine into the peritoneum or outflow from the external ureteral opening were noted. Eleven of 403 patients were suspected of having urinary tract complications. Among the 11 patients, two exhibited damage to the serous and muscular layers of the bladder, while nine had no outflow from the external ureteral opening. Among these nine cases, one patient was found to have right ureteral obstruction, which was attributed to vaginal stump suturing. The sensitivity and specificity for ureteral obstruction detection were 100% and 98.0%, respectively. In the remaining eight patients, no urinary complications could be identified postoperatively. Overall, the rate of bladder injury was 4/403 (0.9%), all of which were repaired intraoperatively, including two cases found by cystoscopy. Ureteral obstruction was identified in 1/403 (0.2%), and the case was due to intraoperative cystoscopy. Postoperatively, ureteral stenosis was observed in 1/403 (0.2%), and urinary tract infection (cystitis: Grade 2 or less) was noted in 6/403 (1.5%). This technique is an effective diagnostic tool with minimal patient burden and is likely to accurately identify ureteral obstruction or bladder injury during surgery.

## Introduction

In a 2015 survey of Gynecologic Oncology Society members in the United States, 97% of respondents had performed robotic surgery, with 75% choosing robotic surgery over laparoscopic surgery for radical hysterectomy and pelvic lymph node dissection for cervical cancer [1]. In Japan, the number of surgeons who experienced more than 50 cases of robotic surgery increased 2.3-fold from the first half of 2021 to the second half of the year [2].

The incidence of iatrogenic urinary tract injury during hysterectomy ranges from 0.4 to 2.9%, with malignant tumors reported to occur more frequently [3–7]. Although surgical procedures rarely result in urinary tract injuries, the consequences of such complications can be detrimental to patient health. However, 62.4% of patients with such injuries were unrecognized intraoperatively, and these patients clearly had an increased risk of re-hospitalization and life-threatening complications [3, 6]. A ureteral fistula tube was needed in 2.3% of patients with recognized ureteral injuries and 23.4% of those with unrecognized injuries. Compared to patients with recognized ureteral injury, patients with unrecognized ureteral injury had a two-fold increased risk of sepsis, 5.9-fold increased risk of ureteral fistula, 23.8-fold increased risk of acute renal failure, and 1.4-fold increased risk of mortality [3].

Hysterectomy in minimally invasive surgery tends to have a higher rate of detection of urinary tract complications, and the usefulness of routine cystoscopy has been reported [8–10]. Most evidence for the routine use of intraoperative cystoscopy to prevent urinary tract complications is limited to patients who have undergone laparotomy or laparoscopy; information on robotic surgery in gynecological surgery is extremely limited [11]. A single study examining robotic surgery failed to determine the effectiveness of cystoscopy in identifying urinary tract complications. This limitation arose from the lack of patients with such complications at facilities that either consistently employ cystoscopy or refrain from its routine use [11].

Since 2017, when we introduced robotic surgery at our facility, we have routinely confirmed the presence or absence of bladder and ureteral complications during surgery using intraoperative cystoscopy with indigo carmine intravenous injection. In this study, we investigated the detection rate and usefulness of routine cystoscopy during gynecological robotic surgery to detect urinary tract complications.

## Patients and methods

### Study design

This retrospective, single-center study created a clinical database to identify the utility of routine cystoscopy in patients who had been treated with robotic surgery between February 2017 and April 2024. The study protocol was approved by the Institutional Review Board of Kagoshima University Graduate School of Medical Sciences (approval # 240153). Participants were informed about the study and offered the opportunity to opt out. Clinical data were collected by reviewing inpatient medical records, and pathological data were collected from histological reports of surgically resected specimens. The primary outcome was the detection rate of bladder and ureteral complications during routine cystoscopy.

Surgical complications were assessed using the Clavien-Dindo Classification v.2.0. Operative time was defined as the time between the start of the skin incision and closure of the incision, following the removal of all ports. Surgeries were performed with the Da Vinci Xi surgical system (Intuitive Surgical Inc., Sunnyvale, CA, USA) and the hinotori Surgical Robot System (Medicaroid Corporation, Kobe, Japan) at the Kagoshima University Hospital. Surgeries were performed by four gynecologic oncologists, each with robot system certification.

### Details of the commonly performed intraoperative cystoscopy technique

After hysterectomy, both sides of the vaginal stump were sutured using a single suture, and the middle part was sutured using a barbed suture (Stratafix Spiral^®^), after which indigo carmine (indigotindisulfonate sodium) was intravenously injected. Upon suturing completion, the area of the peritoneum that appeared thin or ruptured, as revealed by light passing through the cystoscope, was visually inspected. Leakage of indigo carmine into the peritoneum was checked and outflow from the external urethral meatus via the cystoscope was confirmed. In this study, we defined these techniques as routine cystoscopy.

## Results

**Fig. 1:**
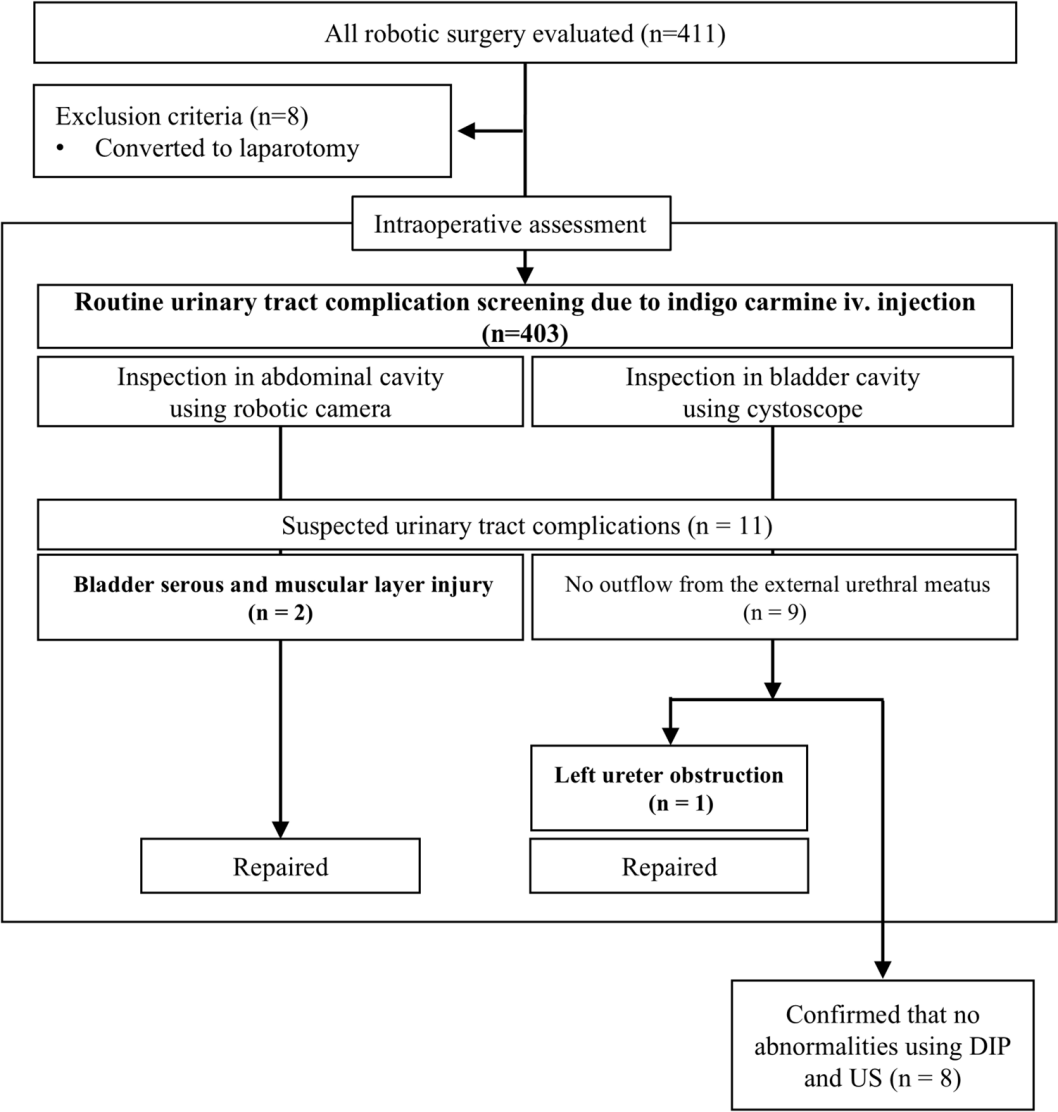
Flowchart summarizing the retrospective analysis. Abbreviation: *iv* intravenous, *DIP* drip infusion pyelography, *US* ultrasound

During the study period, cystoscopy was performed as a standard procedure for all robotic surgeries undertaken in our department. A research flowchart is shown in Fig. 1. Eight patients who converted to laparotomy were excluded, and a total of 403 patients were finally analyzed. The characteristics of 403 patients who underwent routine cystoscopy are listed in Table 1. The median age was 62 years, median body mass index (BMI) was 26.1, and endometrial cancer was the most common disease. No cancer patients were preoperatively suspected to have extrauterine disease.

**Table 1:** Clinical background of the study include 403 patients in gynecological robotic surgery

		n = 403	
^a^Age (range)		62	(28–89)
^a^Height (range)		155.4	(135–172)
^a^Body weight (range)		63.5	(34–135)
^a^BMI (range)		26.1	(13.8–50.1)
Disease			
Endometrial cancer		263	(65.3%)
Cervical cancer		19	(4.7%)
Pelvic organ prolapse		83	(20.6%)
Atypical endometrial hyperplasia		12	(3.0%)
Myoma		7	(1.7%)
Adenomyosis		7	(1.7%)
Squamous intraepithelial lesion		6	(1.5%)
Endometrial polyp		6	(1.5%)
Treatment			
Simple hysterectomy±BSO		40	(9.9%)
Simple hysterectomy±BSO±RPLD or LNBx(SNNS)		258	(64.0%)
Radical hysterectomy(Semi)±BSO±RPLD or LNBx(SNNS)		13	(3.2%)
Radical trachelectomy(Semi)±BSO±RPLD or LNBx(SNNS)		9	(2.3%)
†Sacrocolpopexy		83	(20.6%)
Robotic system			
da Vinci Xi^®^		346	(85.9%)
Hinotori^TM^		57	(14.1%)
^a^Blood loss, ml (range)		25	(0–1125)
^a^Operative times (range)		225	(81–834)
^a^Console/cockpit time (range)		163	(53–636)

^a^All data were indicated in median value

^b^In our facility, surgery is combined with a hysterectomy

*BMI* body mass index, *BSO* bilateral salpingo-oophorectomy, *RPLD* retroperitoneal lymphadenectomy, *LNBx* lymph node biopsy, *SNNS* sentinel navigation surgery

**Fig. 2:**
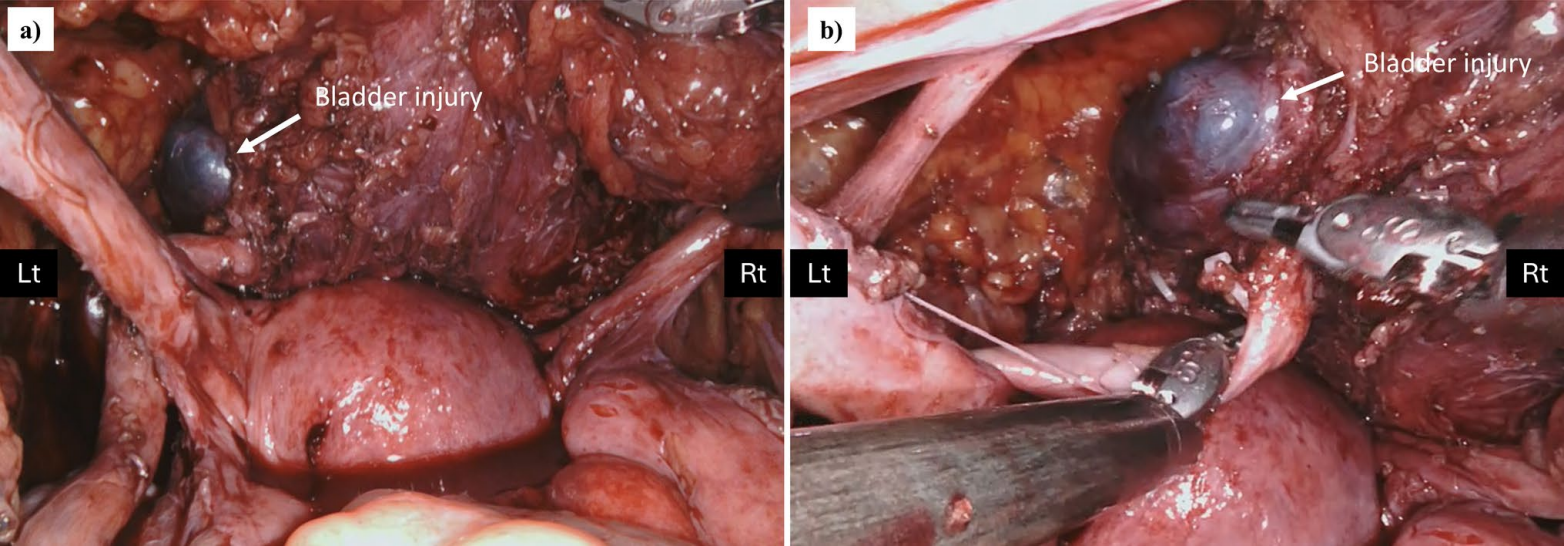
Photographs of bladder serous and muscular layer injuries in Patient No. 1. **a** Bladder mucosal bulging near the left ureteral transition zone during routine cystoscopy after robotic radical trachelectomy. **b** Enlarged image of the bladder injury site

Using our routine cystoscopy procedure, 11 patients (2.7%) were suspected of having urinary tract complications. Among them, three patients were ultimately diagnosed intraoperatively with urinary tract complications, and the remaining eight were assessed as intact. The details of the clinical courses of the three patients are shown in Table 2. Two of the three patients had injuries to the serous and muscular layers of the bladder (Patient No. 1 and 2). A photograph of the bladder mucosa bulging into the abdominal cavity in Patient No 1 is shown in Fig. 2.

**Table 2:** The clinical course of the three patients with suspected urinary tract complications

Patient no.	Injury	Age (y)	Disease	Robot	Treatment	Operation times (min)	Blood loss (m)	The detail of the urinary tract injury
1	Bladder serous and muscular layer injury	33	Cervical ca, FIGO stage 1B1	Da Vinci Xi^®^	Radical trachelectomy+BSO+SNNS	741	895	The uterus is normal size, the right ovary is firmly attached to the posterior lobe of the broad mesentery, the posterior uterus and rectum are extensively attached, and the Douglas fossa is closed. A bulge of the bladder mucosa, probably due to bladder muscle layer injury near the left vesicoureteral transition area, was observed and repaired with 3-0 Vicryl with muscle layer and serosal suture. The patient underwent urethral catheter removal on the sixth postoperative course was uneventful.
2	Bladder serous and muscular layer injury	69	Pelvic organ prolapse, stage II	Da Vinci^®^ Xi	Sacrocolpopexy+hysterectomy+BSO	347	5	There appeared to be no leakage in the bladder wall above the vaginal stump, but thinning was observed, so the bladder wall was reinforced and repaired with absorbable sutures.
3	Left ureter obstruction	56	Endometrial ca, FIGO stage 1A	Hinotori^TM^	Total extrafascial hysterectomy+BSO+SNNS	222	39	No adhesions were observed in the abdominal cavity. Indigo carmine outflow from the right ureteral opening was observed, but no outflow from the left was indicated. A portion of the ureter and bladder wall near the vesicoureteral transition area was entrapped in suture threads of left side vaginal stump and the sutures were removed and the vaginal cuff was re-sutured. The urethral catheter was removed on the sixth postoperative day. No complications occurred, and renal function was unchanged from preoperatively.

*BMI* body mass index, *FIGO* International Federation of Gynecology and Obstetrics, *BSO* bilateral salpingo-oophorectomy, *SNNS* sentinel navigation surgery

**Fig. 3:**
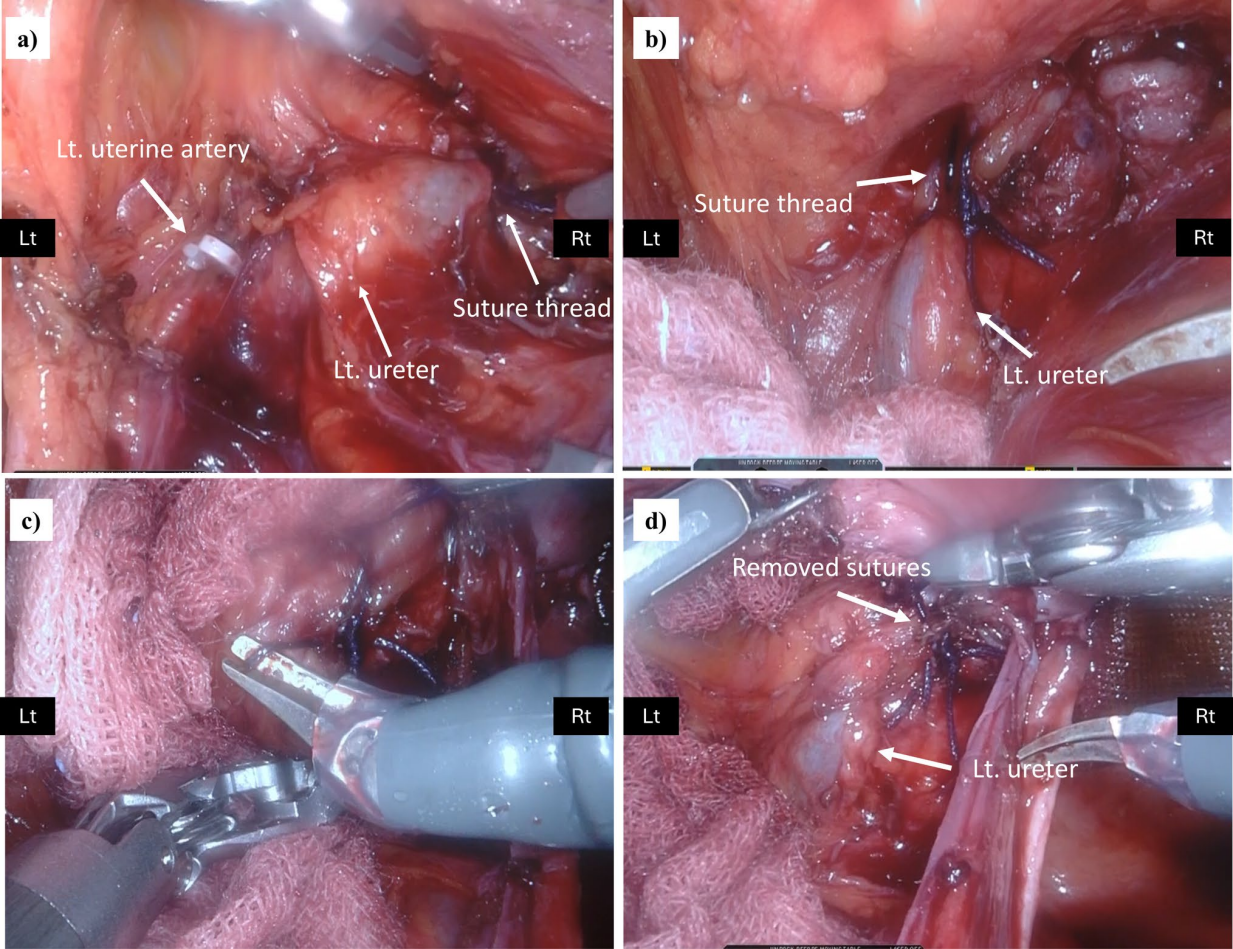
Photographs of the left ureteral obstruction in Patient No.3. **a** The left ureter near the vesicoureteral transition area was entrapped by left-side vaginal stump suture threads, and **b** an enlarged image of the left ureteral obstruction portion. **c** The suture threads were removed and **d** the left ureteral obstruction was resolved

Nine out of 11 patients who were suspected of having urinary tract complications showed no visible urine flow from one of their ureteral orifices during cystoscopic evaluation. None of the patients exhibited an absence of urine flow from both ureteral orifices. These patients underwent a careful examination of the abdominal cavity, and one (0.25%) developed a left ureteral obstruction caused by vaginal cuff sutures (Patient No. 3) (Fig. 3). In our study, the sensitivity and specificity of routine cystoscopy for ureteral obstruction were 100 and 98.0%, respectively. Of the eight patients with no outflow from the ureteral opening and no intraoperative urinary complications that could be identified intraoperatively after close examination, all received an intraperitoneal drain. Postoperatively, none of the patients had urine drainage from the intraperitoneal drain or elevated serum creatinine levels. All the patients underwent ultrasonography or drip infusion pyelography to identify hydronephrosis, which revealed no abnormalities. No ureteral stent insertion or ureteral diverticulum due to heat injury were present.

All the urinary tract complications in the study population are shown in Table 3. Overall, the detection rate for urinary tract complications with routine cystoscopy were 3/403 (0.7%). Of the four patients with intraoperative bladder complications, two underwent repair before routine cystoscopy. Routine cystoscopy failed to identify patients with ureteral stenosis following surgery, possibly because of postoperative adhesions. The rate of urinary tract infections requiring antibiotic administration was 1.5%; all cases were mild with no patients requiring prolonged hospitalization.

**Table 3:** Urinary tract major complications of the 403 patients who underwent robotic surgery

	Total number of patients (Found by routine cystoscopy)
Intraoperative complications	
Bladder injury	4 (2)
Right ureteral obstruction	1 (1)
Right renal artery injury	1
Postoperative complications	
Late ureteral stenosis	1
Urinary tract infection (cystitis: Grade 2 ≤)	6

## Discussion

In our clinical setting, cystoscopy has been routinely performed in all cases since the introduction of robotic surgery. Furthermore, all intraoperative urinary tract complications were detected intraoperatively, including two cases of bladder injury and a patient with ureteral obstruction that was not recognized before routine cystoscopy during the surgery.

More than half of iatrogenic ureteral injuries are associated with gynecologic surgery, accounting for 17% of non-obstetric lawsuits against gynecologists in the United States [3, 12]. The rates of rehospitalization after hysterectomy (baseline: 5.7%) and the need for nephrostomy insertion were 13.4 and 2.3% after recognized injury, and 67.3 and 23.4% after unrecognized ureteral injury, respectively. Unrecognized ureteral injuries significantly increase the risk of acute renal failure and death [3]. Undiagnosed ureteral injuries that require readmission and delayed open repair cost 1.72 times more than those detected in women who undergo immediate repair. In addition, these costs are 2.29 times higher than those associated with patients who undergo surgery without ureteral complication [13]. To avoid such worst-case outcomes, it is important too recognize ureteral injury as early as possible intraoperatively to allow for prompt repair [14].

Regarding iatrogenic ureteral complications, over 90 percent were located in the lower third of the ureter, and intravenous administration of indigo carmine during surgery has been found to be useful for detecting damage to the lower urinary tract [6, 15]. In urogynecologic surgeries, 2.9% of cases required intervention for urinary tract damage detected by cystoscopy with intravenous indigo carmine administration, and no cases were identified after surgery [6].

A review of ureteral complications during benign gynecologic surgery, including robotic surgery, found that intraoperative ureteral and bladder complication rates were significantly higher when intraoperative cystoscopy was routinely performed, with ureteral injury and bladder complication rates of 0.3 and 0.8%, respectively. In contrast, the postoperative detection rate of ureteral complications was low (0.07 vs. 0.16%) when cystoscopy was performed, but there was no difference in the postoperative detection rate of bladder complications (0.1 vs. 0.08%) with cystoscopy [7]. There was no significant reduction in the rate of delayed lower urinary tract complications between patients who underwent cystoscopy and those who did not (0.27 vs. 0.24%, p = 0.64), even in a large cohort that included around half of the cases being performed using laparoscopic or robotic techniques [16].

Factors that have a significant impact on the recovery of renal function after ureteral obstruction include the duration of the obstruction, patient’s age, thickness of the renal cortex, renal blood flow and perfusion, and degree of hydronephrosis [17–19]. Delaying relief of obstructive ureteral injury by over two weeks leads to decreased long-term renal function and increased risk of hypertension or preexisting hypertension [17]. In our study, three patients with ureteral or bladder injuries were identified during surgery prior the routine cystoscopy. However, these injuries did not lead to postoperative complications, suggesting that they may have been incidentally repaired during the surgery, thus preventing the need for rehospitalization and having no impact on the patients' quality of life. We experienced two cases of bladder muscle layer rupture; these can sometimes lead to bladder rupture, and symptoms including abdominal pain, abdominal distension, dysuria, oliguria or anuria, and fever are occasionally nonspecific, leading to misdiagnosis or delayed diagnosis of acute abdomen, inflammatory gastrointestinal disease, and other conditions, which can be fatal in some patients [20]. The diagnosis of postoperative bladder minor leaks is sometimes difficult. A routine trauma CT scan may not depict bladder rupture in all patients, and a delayed CT scan may help determine which patients require subsequent cystography. Retrograde cystography is the most reliable test; however, its use is often limited [21–23]. Blood urea and creatinine levels may support the diagnosis in difficult clinical situations [24].

In ureteral obstruction, it is safe to release the ureteral ligation within 24 h, and complete recovery from damage can be expected in in vivo experiments; however, the risk of complications such as hydronephrosis increases with ligation for more than 48 h; therefore, ligations should be released as soon as possible [25]. Although the ureter was ligated intraoperatively in Patient No. 3 in this study, the suture was released immediately after ligation, so there was no decline in renal function or subsequent renal complications. Patients without a routine cystoscope to detect the obstruction postoperatively might have required vesicoureteral neoanastomosis.

Regarding complications in routine cystoscopy during robotic surgery, there is a potential for a high incidence of urinary tract infections (UTI) [26, 27], prolonged surgical times [11, 26], and rare cases of anaphylactic reactions to indigo carmine injection [28]. In our study, the risk of UTI requiring antibiotic administration was low under intraoperative prophylactic antibiotic administration, and routine cystoscopy could be performed without interrupting the robotic procedure, with little risk of extending surgery time. We did not observe any anaphylactic reactions due to indigo carmine.

We searched the PubMed database for original articles published over the past 10 years, in accordance with the original article on mainly laparoscopic surgery in a systematic review published in 2014 that estimated the incidence of urinary tract injury associated with minimally invasive surgery, including robotic surgery [11, 16, 26, 27, 29]. The keywords used were “ureter or ureteral or urethra or urethral” or “bladder” or “urinary tract” and “injury” and “robotic” and “gynecology” and “hysterectomy” and “cystoscopy.” Six studies were identified in the literature and are listed in Table 4; in two of the studies no complication rates were reported. Two of the remaining four studies lacked patients with urinary tract injuries and were therefore not adequately evaluated [11, 29]. The final two studies used the national quality database, which is a multicenter data collection system, and the frequency of intraoperative urinary tract complications could not be confirmed [16, 26]. Therefore, to date, our report is the only one to have accurately demonstrated the rate of urinary tract complications using a cystoscope in robotic surgery.

**Table 4:** Literatures on cystoscopy detection of urinary tract complication in gynecological robotic surgery

Authors	Year (y)	Trial	Laparotomy/Laparoscopy/robot	Malignant/Benign	Number of patients	BMI (Median)	Operation times (min, Median)	Blood loss (ml, Median)	Urinary tract complication rates	
									Diagnosed by cystoscopy	Diagnosed post-operatively
Nguyen et al [11]	2014	Retrospective	Robot	Malignant/Benign	249	33	180–360	68.3–101.6	0%	0%
Barber et al [16]	2019	Retrospective	Laparotomy/Laparoscopy/robot	Benign	39529 (national quality database)	N/A	N/A	N/A	N/A	Only laparoscopy/robot: cystoscopy: 0.20% without cystoscopy: 0.18%
Polan et al [26]	2022	Retrospective	Laparotomy/Laparoscopy/robot	Malignant/Benign	33355 (national quality database)	N/A	N/A	N/A	N/A	Only laparoscopy/robot: cystoscopy: 0.28% without cystoscopy: 0.22%
Galhotra et al [29]	2023	Prospective	Laparoscopy and robot	Benign	91	32	117–124	26.5–50	0%	0%
Current study	2024	Retrospective	Robot	Malignant/Benign	403	26.1	225	25	0.007% (three patients)	0.005% (two patients)

*BMI* body mass index, *N/A* not applicable

The significance of the routine intraoperative cystoscopy procedure in our department is that it allows close examination of bladder wall complications, in addition to detecting potential ureteral obstruction. The procedure can be performed by an assistant after handling a vaginal cuff, and a simple cystoscope can be used without additional staffing. Indigo carmine is frequently used in routine clinical practice and is inexpensive. The rare complication of an anaphylactic reaction is not an excessive concern as patients are under general anesthesia.

Postoperative ureteral obstruction may not become apparent unless an abnormality is identified during intra-abdominal exploration, even if ureteral outflow cannot be confirmed during surgery. Complications in the bladder wall could also be clearly observed based on permeability of the light source and the blue indigo carmine dye that could be seen through the cystoscope. The limitations of our study are that it was retrospective, the primary disease varied, and the number of events was insufficient for a definitive evaluation.

## Conclusion

In conclusion, our study provides significant data on intraoperative routine cystoscopy for urinary tract complications in a single institution. This technique is a useful diagnostic modality with minimal burden to the patient and is likely to accurately identify ureteral obstruction and bladder injury intraoperatively. Further extensive clinical trials to establish standard procedures are needed.

## Data Availability

The data for this study are shown in tables and figures; no other datasets were generated or analyzed during the current study.
